# Rabies Virus in Raccoons, Ohio, 2004

**DOI:** 10.3201/eid1404.070972

**Published:** 2008-04

**Authors:** J. Caroline Henderson, Roman Biek, Cathleen A. Hanlon, Scott O'Dee, Leslie A. Real

**Affiliations:** * Emory University, Atlanta, Georgia, USA; † Kansas State University, Manhattan, Kansas, USA; ‡ Ohio Department of Health, Reynoldsburg, Ohio, USA; 1 Current affiliation: Centers for Disease Control and Prevention, Atlanta, Georgia, USA.; 2 Current affiliation: University of Glasgow, Glasgow, Scotland, UK.

**Keywords:** Rabies virus, wild animals, raccoon, genetic markers, vaccination, dispatch

## Abstract

In 2004, the raccoon rabies virus variant emerged in Ohio beyond an area where oral rabies vaccine had been distributed to prevent westward spread of this variant. Our genetic investigation indicates that this outbreak may have begun several years before 2004 and may have originated within the vaccination zone.

Several wild carnivorous mammals may be competent zoonotic reservoirs for rabies viruses (*1*). Similar to how parenteral vaccination has contributed to control and elimination of rabies in dogs, effective oral rabies vaccines and application methods for wildlife species, most notably the red fox (*Vulpes vulpes*), have led to regional containment and elimination of the rabies virus variants associated with this species in large parts of Canada and Europe (*2*). The first step toward reducing the size of areas in which rabies is enzootically transmitted is containment of its regional spread. Understanding the conditions under which containment of wildlife rabies can reliably be achieved will facilitate the long-term goal of eliminating particular rabies virus variants from their respective reservoir species.

During the late 1970s, the range of a raccoon (*Procyon lotor*)–specific rabies virus variant (RRV) expanded substantially from the historically affected southeastern United States to the currently affected eastern North America (*3*). In 1996, to contain westward expansion of this variant, oral rabies vaccine (ORV) was distributed in Ohio. The ORV strategy includes distributing bait containing a vaccinia-rabies glycoprotein recombinant vaccine (*4*) while taking advantage of physiogeographic impediments to rabies transmission, such as mountains, rivers, and major highways to create a barrier 50 km–150 km wide between unaffected and enzootic areas.

During 1999–2004, ORV had apparently limited further spread of the virus (*5*) (Figure 1). However, in July 2004, RRV was diagnosed in a raccoon northwest of the ORV zone in Lake County, Ohio. As of December 2005, enhanced surveillance had detected 77 rabid raccoons in Lake County and 2 adjacent counties (Geauga and Cuyahoga) (Figures 1 and 2, panel** A**). These detections raised the question whether current ORV and surveillance strategies are sufficient for containment and reaching the long-term goal of regional elimination of RRV. We used molecular analyses to gain insight into the factors and possible raccoon source populations associated with the breach of the ORV zone in Ohio.

**Figure 1:**
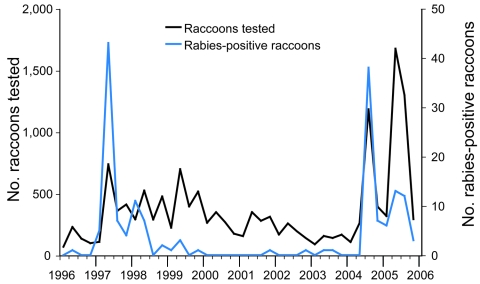
Raccoon rabies surveillance efforts in Ohio, 1996–2005. Data were aggregated at 3-month intervals.

**Figure 2:**
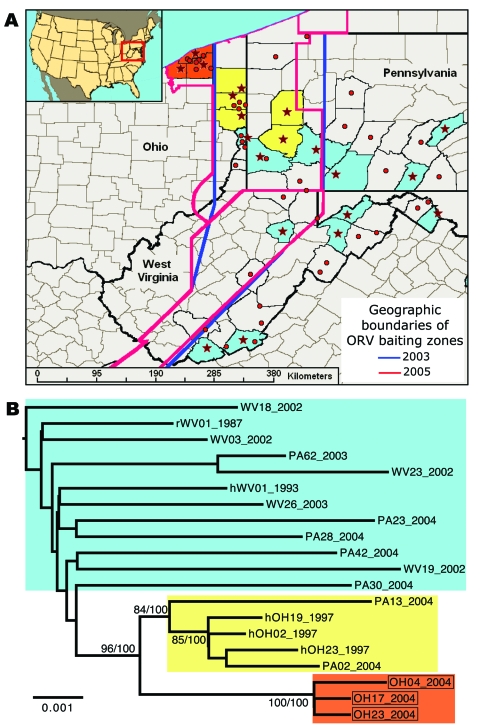
Spatial and genetic distribution of sequences of the raccoon rabies virus variant (RRV) from the 2004 Ohio outbreak relative to virus found in neighboring areas. A) Distribution of RRV samples included in phylogenetic analysis of G and N gene sequences (stars) or G sequences only (circles). Colors reflect phylogenetic groups as shown in panel B. B) Maximum-likelihood tree of concatenated G and N gene sequences of RRV sampled in or near Ohio, 1987–2004. Samples from the 2004 outbreak are boxed. Bootstrap values and corresponding Bayesian posterior values (% for both) are shown for key nodes. Tree was rooted by using RRV G and N sequences from a Florida raccoon (not shown). ORV, oral rabies vaccine. Scale bar = nucleotide substitutions per site.

## The Study

Viral RNA was extracted as described (*6*) from brain tissue of 67 rabid raccoons. Samples came from raccoons in Ohio (1996 [n = 9] and 2004 [n = 10] outbreaks) and the neighboring states of Pennsylvania (2003–2004 [n = 21] and West Virginia (1987–2004 [n = 27]) (Appendix Table). We amplified a 1,345-nt portion of the glycoprotein gene (G) and, for a smaller subset of samples (n = 20), the complete nucleoprotein gene (N) (1,416 nt,) (see [6] for primers and conditions). Sequences from a Florida raccoon (G, U27216; N, U27220) were included as an outgroup. When sequence data for G and N had been obtained, sequences were concatenated. After alignment, appropriate evolutionary models (*7*) were found for phylogenetic estimation by using maximum-likelihood and Bayesian approaches (*8*,*9*). Maximum-likelihood trees were constructed by using heuristic searches, and node support was assessed with 1,000 bootstrap replicates under the distance criterion with maximum-likelihood model settings. Bayesian estimation was performed with 2 runs of 6 million samples each and a sampling frequency of 1,000; the first 1,000 samples were discarded as burn-in.

A Bayesian molecular clock–based method (*10*) was used to estimate when the 2004 RRV lineage had started to diversify. To estimate evolutionary rates, we included 3 raccoon rabies sequences isolated during the larger Atlantic coast epizootic of 1982–1984. Analyses were run for 10 million steps after a burn-in period of 1 million under an exponential growth model; alternative demographic models produced equivalent results (data not shown).

According to the combined G and N data, the phylogenetic analyses showed that the 2004 Ohio outbreak was caused by a distinct RRV lineage that had limited diversity (Figure 2, panel** B**, **red**), which suggests a single-source introduction into Ohio. The 2004 lineage was not a direct descendent of any previously sampled lineages, but it shared a common ancestor with another lineage (Figure 2, panel** B**, **yellow**) that contained the viruses responsible for the 1996 Ohio outbreak along with contemporary viruses from western Pennsylvania. No members of either of these lineages had been found east of the ORV barrier (Figure 2, panel** A**), an area dominated by a different group of viruses (Figure 2, panel** B**, **blue**). The same result was obtained when the larger dataset based on G data only was analyzed (Appendix Figure) and when we included RRV sequences from throughout eastern North America (data not shown). This finding suggests that the virus associated with the 2004 outbreak in Ohio most likely originated within the ORV zone.

Temporal estimates further indicated that all viruses sampled in the recent Ohio outbreak had started to diversify at least 3 years before 2004. The estimated dates associated with the most recent common ancestor were 1998 (highest posterior density interval 1993–2001) for the concatenated G and N data and 1995 (highest posterior density interval 1990–2000) for G data only.

## Conclusions

Our findings imply that RRV had been circulating undetected among raccoons in the ORV zone, and possibly beyond it, for several years before its detection in 2004. These findings have important implications for the control of wildlife rabies in raccoons through ORV. First, the genetic analyses do not point to a long-distance transmission event to Ohio but rather suggest that the virus was indigenous to the region. In view of potential continued transmission events within the current ORV zone, widening the ORV corridor likely will not prevent such transmission and further spread. Second, the findings suggest that RRV may be able to persist within the ORV zone for several years and thus provide continued risk for eventual spread into unvaccinated raccoon populations. Insufficient levels of immunization among the overall population could contribute to this situation. However, spatial variation in the level of immunization or random fluctuations in the number of infected animals may also enable the virus to persist in parts of the ORV zone. Third, the level of surveillance needed to detect RRV when transmission frequency is low is unclear.

Our results indicate that the virus had been present within Ohio for several years when surveillance efforts were relatively low; from January 2000 through June 2004, an average of 71 raccoons were tested each month compared with an average of 139 per month during 1997–1999 (Figure 1). Therefore, the critical question is: at what point would the marginal cost of increased surveillance leading to earlier detection have outweighed the cost associated with controlling the 2004 outbreak? To develop the most cost-effective strategy for containment and ultimate elimination of rabies among raccoons, further analyses should aim at quantifying this trade-off.

## Supplementary Material

Appendix Table Samples and GenBank accession nos. used in this study*

Appendix Figure Maximum-likelihood tree of 67 partial G gene sequences of raccoon rabies virus sampled in or near Ohio, 1987–2004. Sequences from the 2004 outbreak are shown in boldface. Bootstrap values and corresponding Bayesian posterior values (both as percentages) are shown for key nodes. Tree was rooted using a raccoon rabies virus sequence from a Florida raccoon (not shown). Numbers following taxa names indicate the time of sampling, which is expressed relative to the year 1900 (i.e., '102.6' represents June 10 of the year 2002). See Figure 2 for further details.

## References

[1] Jackson AC, Wunner WH, eds. Rabies. 2nd ed. London: Academic Press; 2007.

[2] Rupprecht CE, Hanlon CA, Slate D. Oral vaccination of wildlife against rabies: opportunities and challenges in prevention and control. Dev Biol (Basel). 2004;119:173–84. 15742629

[3] Rupprecht CE, Smith JS. Raccoon rabies: the re-emergence of an epizootic in a densely populated area. In: Seminars in virology; 1994. p. 155–64.

[4] Hanlon CA, Niezgoda M, Hamir AN, Schumacher C, Koprowski H, Rupprecht CE. First North American field release of a vaccinia-rabies glycoprotein recombinant virus. J Wildl Dis. 1998;34:228–39. 9577769 10.7589/0090-3558-34.2.228

[5] Slate D, Rupprecht CE, Rooney JA, Donovan D, Lein DH, Chipman RB. Status of oral rabies vaccination in wild carnivores in the United States. Virus Res. 2005;111:68–76. 10.1016/j.virusres.2005.03.012 15896404

[6] Biek R, Henderson JC, Waller LA, Rupprecht CE, Real LA. A high-resolution genetic signature of demographic and spatial expansion in epizootic rabies virus. Proc Natl Acad Sci U S A. 2007;104:7993–8. 10.1073/pnas.0700741104 17470818 PMC1876560

[7] Posada D, Crandall KA. MODELTEST: testing the model of DNA substitution. Bioinformatics. 1998;14:817–8. 10.1093/bioinformatics/14.9.817 9918953

[8] Ronquist F, Huelsenbeck JP. MrBayes 3: Bayesian phylogenetic inference under mixed models. Bioinformatics. 2003;19:1572–4. 10.1093/bioinformatics/btg180 12912839

[9] Swofford DL. PAUP* (Phylogenetic analysis using parsimony) (*and other methods). Version 4.0b10. Sunderland (MA): Sinauer Associates; 2002.

[10] Drummond AJ, Rambaut A. BEAST (Bayesian evolutionary analysis by sampling trees). BMC Evol Biol. 2007;7:214. 10.1186/1471-2148-7-214 17996036 PMC2247476

