# Supplementary material for: Rabies Virus in Raccoons, Ohio, 2004
Source: Emerg Infect Dis. 2008 Apr;14(4):650–2. doi: 10.3201/eid1404.070972 (PMC2570922; doi:10.3201/eid1404.070972)
Supplement: Appendix Table — Samples and GenBank accession nos. used in this study* [file 07-0972_appT.pdf]

Appendix Table. Samples and GenBank accession numbers used in this study

GenBank no.	Prep ID	SM	State	County	Date	Gene
DQ886040	hWV01N	SM1711	WV	Hancock	1993 Mar 1	N
DQ886046	PA13N	PA0407093	PA	Butler	2004 Sep 9	N
DQ886047	PA28N	PA0401719	PA	Mifflin	2004 Jul 13	N
DQ886070	rWV01N	SM1845	WV	Grant	1987 Aug 1	N
DQ886072	WV03N	WV278	WV	Monroe	2002 Aug 7	N
DQ888333	hWV01G	SM1711	WV	Hancock	1993 Mar 1	G
DQ888339	PA13G	PA0407093	PA	Butler	2004 Sep 14	G
DQ888340	PA28G	PA0401719	PA	Mifflin	2004 Jul 13	G
DQ888363	rWV01G	SM1845	WV	Grant	1987 Aug 1	G
DQ888365	WV03G	WV278	WV	Monroe	2002 Aug 7	G
DQ888367	WV08G	WV390	WV	Pendleton	2002 Aug 19	G
DQ888368	WV14G	WV465	WV	Hardy	2002 Aug 30	G
EF508133	PA23G	PA0406025	PA	Fulton	2004 Aug 26	G
EF508134	PA30G	SM6372	PA	Somerset	2004 Mar 24	G
EF508135	PA62G	3R3532	PA	Washington	2003 Apr 8	G
EF508136	WV19G	WV549	WV	Jefferson	2002 Sep 17	G
EF508137	WV23G	WV569	WV	Mineral	2002 Sep 20	G
EF508138	WV26G	SM6487	WV	Barbour	2003 Oct 29	G
EF508139	PA23N	PA0406025	PA	Fulton	2004 Aug 26	N
EF508140	PA30N	SM6372	PA	Somerset	2004 Mar 24	N
EF508141	WV19N	WV549	WV	Jefferson	2002 Sep 17	N
EF508142	WV23N	WV569	WV	Mineral	2002 Sep 20	N
EF508143	WV26N	SM6487	WV	Barbour	2003 Oct 29	N
EF508144	PA62N	3R3532	PA	Washington	2003 Apr 8	N
EU003121	hOH02N	SM3448	OH	Columbiana	1997 May 1	N
EU003122	hOH19N	SM3464	OH	Mahoning	1997 May 13	N
EU003123	hOH23N	SM3449	OH	Trumbull	1997 May 1	N
EU003124	OH04N	SM6285	OH	Cuyahoga	2004 Aug 6	N
EU003125	OH17N	SM6311	OH	Geauga	2004 Aug 30	N
EU003126	OH23N	SM6326	OH	Lake	2004 Sep 15	N
EU003127	PA02N	PA2465023_4	PA	Allegheny	2004 May 12	N
EU003128	PA42N	PA2465023_8	PA	Westmoreland	2004 May 12	N
EU003129	WV18N	WV546	WV	Mercer	2002 Sep 16	N
EU006544	hOH23G	SM3449	OH	Trumbull	1997 May 1	G
EU006545	hOH02G	SM3448	OH	Columbiana	1997 May 1	G
EU006546	hOH04G	SM3454	OH	Columbiana	1997 Apr 30	G
EU006547	hOH05G	SM3222	OH	Mahoning	1996 May 1	G
EU006548	hOH07G	SM3380	OH	Mahoning	1997 Mar 28	G
EU006549	hOH19G	SM3464	OH	Mahoning	1997 May 13	G
EU006550	hOH20G	SM3465	OH	Mahoning	1997 May 13	G
EU006551	hOH21G	SM3466	OH	Mahoning	1997 May 13	G
EU006552	OH17G	SM6311	OH	Geauga	2004 Aug 30	G
EU006553	OH01G	OH30409	OH	Lake	2004 Jul 30	G
EU006554	OH04G	SM6285	OH	Cuyahoga	2004 Aug 6	G
EU006555	OH10G	SM6291	OH	Geauga	2004 Aug 6	G
EU006556	OH11G	SM6292	OH	Geauga	2004 Aug 7	G
EU006557	OH12G	SM6294	OH	Lake	2004 Aug 9	G
EU006558	OH15G	SM6308	OH	Geauga	2004 Aug 23	G
EU006559	OH16G	SM6309	OH	Geauga	2004 Aug 27	G
EU006560	PA22G	PA0407462	PA	Franklin	2004 Sep 20	G
EU006561	OH18G	SM6311	OH	Lake	2004 Aug 30	G
EU006562	OH23G	SM6326	OH	Lake	2004 Sep 15	G
EU006563	PA02G	PA2465023_4	PA	Allegheny	2004 May 12	G
EU006564	PA04G	PA0402143_3	PA	Allegheny	2004 Jul 16	G
EU006565	PA10G	SM6371	PA	Bedford	2004 Mar 7	G
EU006566	PA15G	PA0406341	PA	Cambria	2004 Aug 31	G
EU006567	PA19G	SM6377	PA	Fayette	2004 Aug 30	G
EU006568	PA55G	3R3513	PA	Crawford	2003 Apr 11	G
EU006569	PA25G	PA0405472	PA	Huntingdon	2004 Aug 19	G
EU006570	PA26G	PA0400421_9	PA	Indiana	2004 Jun 28	G
EU006571	PA27G	PA0407458	PA	Jefferson	2004 Sep 20	G
EU006572	PA32G	PA0401161	PA	Washington	2004 Jul 8	G
EU006573	PA33G	PA0402026_2	PA	Washington	2004 Jul 15	G
EU006574	PA38G	SM6376	PA	Westmoreland	2004 Apr 13	G
EU006575	PA42G	PA2465023_8	PA	Westmoreland	2004 May 12	G
EU006576	WV16G	WV531	WV	Greenbrier	2002 Sep 12	G
EU006577	PA57G	3R3717	PA	Erie	2003 May 6	G
EU006578	PA58G	3R4497	PA	Fayette	2004 Jul 2	G

EU006579	WV01G	WV210	WV	Raleigh	2002 Jul 30	G
EU006580	WV06G	WV384	WV	Hardy	2002 Aug 19	G
EU006581	WV07G	WV385	WV	Berkeley	2002 Aug 19	G
EU006582	WV09G	WV400	WV	Hancock	2002 Aug 20	G
EU006583	WV11G	WV410	WV	Webster	2002 Aug 21	G
EU006584	WV30G	SM5998	WV	Hancock	2003 Jul 16	G
EU006585	WV18G	WV546	WV	Mercer	2002 Sep 16	G
EU006586	WV21G	WV552	WV	Pendleton	2002 Sep 17	G
EU006587	WV24G	WV581	WV	Monongalia	2002 Sep 24	G
EU006588	WV25G	WV1014	WV	Berkeley	2002 Dec 23	G
EU006589	WV27G	SM6483	WV	Brooke	2003 Sep 10	G
EU006590	WV28G	SM5995	WV	Greenbrier	2003 Jul 1	G
EU006591	WV48G	SM6002	WV	Preston	2003 Aug 8	G
EU006592	WV37G	SM6484	WV	Monongalia	2003 Sep 15	G
EU006593	WV40G	SM5993	WV	Monroe	2003 Jun 19	G
EU006594	WV42G	SM6489	WV	Monroe	2004 Feb 10	G
EU006595	WV45G	SM5977	WV	Ohio	2003 Mar 11	G
EU006596	WV46G	SM5980	WV	Ohio	2003 Apr 3	G
EU006597	WV47G	SM5973	WV	Preston	2003 Feb 4	G
