# Supplementary figures and images for: Rabies Virus in Raccoons, Ohio, 2004
Source: Emerg Infect Dis. 2008 Apr;14(4):650–2. doi: 10.3201/eid1404.070972 (PMC2570922; doi:10.3201/eid1404.070972)

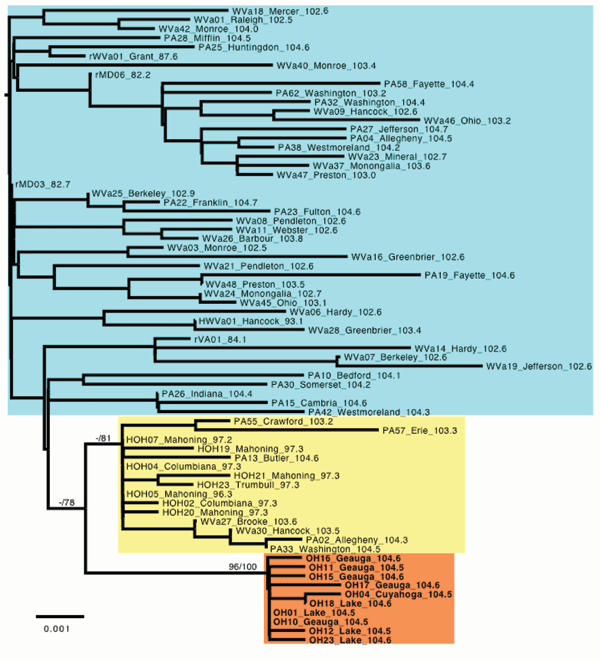

Supplement: Appendix Figure — Maximum-likelihood tree of 67 partial G gene sequences of raccoon rabies virus sampled in or near Ohio, 1987–2004. Sequences from the 2004 outbreak are shown in boldface. Bootstrap values and corresponding Bayesian posterior values (both as percentages) are shown for key nodes. Tree was rooted using a raccoon rabies virus sequence from a Florida raccoon (not shown). Numbers following taxa names indicate the time of sampling, which is expressed relative to the year 1900 (i.e., '102.6' represents June 10 of the year 2002). See Figure 2 for further details. [file 07-0972_app.gif]
